# Cross-Talk between Mucosal-Associated Invariant T, Natural Killer, and Natural Killer T Cell Populations is Implicated in the Pathogenesis of Placenta Accreta Spectrum

**DOI:** 10.1007/s10753-023-01799-1

**Published:** 2023-03-31

**Authors:** Omnia El-Badawy, Ahmed M. Abbas, Eman Radwan, Rania Makboul, Areej A. Khamis, Maha Ali, Mai M. Elkabsh, Marwa H. Bakr, Asmaa M. Zahran

**Affiliations:** 1 Department of Medical Microbiology and Immunology, Faculty of Medicine, Assiut University, Assiut, 71515 Egypt; 2 Obstetrics and Gynecology Department, Faculty of Medicine, Assiut University, Assiut, Egypt; 3 Department of Medical Biochemistry, Faculty of Medicine, Assiut University, Assiut, Egypt; 4 Department of Biochemistry, Sphinx University, New Assiut, Assiut, Egypt; 5 Pathology Department, Faculty of Medicine, Assiut University, Assiut, Egypt; 6 Department of Histology and Cell Biology, Faculty of Medicine, Assiut University, Assiut, Egypt; 7 Department of Clinical Pathology, South Egypt Cancer Institute, Assiut University, Assiut, Egypt

**Keywords:** MAIT, NK cells, NKT cells, placenta accreta spectrum, VEGF, ENG, sFLT-1

## Abstract

The study included 32 women with PAS and 20 with normally implanted placenta as a control group. Vascular endothelial cell growth factor (VEGF), Soluble FMS Like Tyrosine Kinase (sFLT-1/sVEGFR1), and Endoglin (ENG) were measured in placenta tissue by ELISA. Granzyme B (GrzB) expression in trophoblastic and stromal mesenchymal cells was evaluated by immunohistochemistry. MAIT, NK, and NKT cells were assessed in blood and placenta by flow cytometry. Alterations were observed in levels of MAIT cells, NK cell subsets, and NKT cells in patients compared with controls. Several significant correlations were detected between these cells and GrzB scores, VEGF, ENG, and sFLT-1 levels. This is the first study analysing these cells in PAS patients and correlating their levels with changes in some angiogenic and antiangiogenic factors implicated in trophoblast invasion and with GrzB distribution in trophoblast and stroma. Interrelation between these cells probably plays an important role in pathogenesis of PAS.

**Supplementary Information:**

The online version contains supplementary material available at 10.1007/s10753-023-01799-1.

## INTRODUCTION

Placenta accreta spectrum (PAS) is a serious obstetric complication caused by extensive invasion of the placental villi through the myometrium [1]. The increasing global incidence of PAS is due to a continuing rise in the cesarean section rate [2]. PAS is a major cause of maternal morbidity and mortality, leading to uncontrolled postpartum hemorrhage, which may inevitably necessitate hysterectomy [3].

PAS is often accompanied by chronic basal inflammation and impaired placental apoptosis, partly elucidating the basic biology of invasive extravillous trophoblasts with abnormal maternal vascular remodeling [4]. The placenta of a normal pregnancy is rich in its unique and diverse microbiome [5]. It is believed that the microbiota modulates the inflammatory responses at the maternal–fetal interface, preventing rejection [6]. Dysbiosis of the placental microbiome prompts a proinflammatory state leading to adverse pregnancy outcomes [7]. The presence of proinflammatory markers that signify a greater risk of PAS suggests the contribution of dysbiosis in the PAS pathogenesis [8].

In comparison with peripheral blood, the intervillous spaces of the term placenta are rich in mucosal-associated invariant T (MAIT) cells [9, 10]. MAIT cells are also detected in decidual tissues [11]. MAIT cells are non-conventional innate-like T cells expressing the semi-invariant T cell receptor alpha chain Vα7.2 (TRAV1-TRAJ33) [12], which recognizes Riboflavin metabolites presented by the major histocompatibility class 1-related molecule (MR1) [12]. Many commensal and pathogenic bacteria and fungi produce these non-peptide MAIT cell ligands [12, 13]. Also, inflammatory molecules, chiefly IL-12 and IL-18, can partly stimulate MAIT cells in a TCR-independent manner [14]. MAIT cells demonstrate an intrinsic effector/memory phenotype. Upon stimulation, they quickly secrete several inflammatory cytokines like tumor necrosis factor-α, (TNF-α), interferon-γ (IFN-γ) and IL-17 [15], in addition to cytotoxic mediators as granzyme B (GrzB) and perforin [16]. Therefore, they need to be under strict control [17].

PAS pathogenesis has not yet been fully understood, and data regarding immune dysregulation in PAS are still lacking. Recent studies reported an association between MAIT cells and microbiota [18, 19]. Intervillous and decidual MAIT cells might have a defensive role by inhibiting bacteria from crossing the feto-maternal barrier [17]. To date, the relation of MAIT cells, natural killer (NK), and NKT cells (NKT) with PAS has not been evaluated. Thus, we aimed to assess the alterations in NK cell subsets, NKT and MAIT cells in placenta and blood of PAS patients and correlate their levels with the changes in some angiogenic and antiangiogenic factors implicated in trophoblast invasion and with GrzB distribution in trophoblast and stroma.

## METHODS

Fifty-two pregnant women in their third trimester, admitted to the Women Health Hospital for elective cesarean section, were enrolled in this case–control study. Thirty-two of them were diagnosed formerly with PAS during routine antenatal ultrasonography (US). The other 20 women had normally implanted placenta, admitted for elective cesarean section due to repeated cesarean sections or malpresentation, and were enrolled as a control group. Patients with recurrent pregnancy loss, intrauterine fetal death, intrauterine growth restriction, intrauterine infections, multiple gestation, major fetal anomalies, Rh isoimmunization, hypertensive disorders with pregnancy, diabetes mellitus, patients with renal or liver diseases, systemic lupus erythematosus (SLE), or other collagen diseases, patients on steroid therapy during a week before the delivery, antiphospholipid syndrome, immunocompromised patients, polyhydramnios, premature rupture of membranes and patients starting uterine contractions were excluded from the study.

### Sample Collections

During the cesarean section, two milliliters of peripheral blood were collected from each participant in addition to several placental bed biopsy specimens at the attachment site to the uterine wall. Biopsies were divided into three parts for flow cytometry, enzyme-linked immunosorbent assay (ELISA), and immunohistochemistry. According to FIGO classification 2019 [20], PAS were categorized into three grades based on the clinical and histological findings: Grade 1: Abnormally adherent placenta (placenta adherenta or accreta), Grade 2: abnormally invasive placenta (Increta), and Grade 3: Abnormally invasive placenta (Percreta).

### Enzyme-Linked Immunosorbent Assay

Tissue was homogenized, and total protein assay was performed using Total Protein Assay Kit (Elabscience, USA). Human Vascular endothelial cell growth factor (VEGF), Soluble FMS Like Tyrosine Kinase (sFLT-1/sVEGFR1), and Endoglin (ENG) were measured in tissue homogenates by ELISA kits purchased from SinogeneClon Biotech Co., China, according to manufacturers' instructions (Catalogs no. SG-10402, SG-10466, and SG-10522 respectively).

### Histopathology and Immunohistochemistry

#### Microscopic Examination

Placenta samples were fixed in 10% formalin for 24 h, dehydrated, cleared, and paraffin-embedded. Sections, 5 μm thick, were cut from paraffin blocks and were stained with hematoxylin and eosin (H&E) for routine histopathologic evaluation.

#### Immunohistochemistry

Tissue sections (5 μm) were deparaffinized, rehydrated in graded alcohol, and transferred to phosphate-buffered saline (PBS; pH7.6). The slides were rinsed twice with PBS, and then endogenous peroxidase was blocked by using 3% hydrogen peroxidase in methanol for 5 min.

Antigen retrieval was performed by immersing the slides in citrate buffer and putting them in the microwave for 15 min. Then the slides were incubated overnight at 4^◦^C with primary antibody for GrzB (mouse polyclonal antibody, bs-1351R, Bioss antibodies) using peroxidase-labeled streptavidin–biotin (LSAB) at a concentration of 1/100. Then diaminobenzidine was applied for 5 min at room temperature. Finally, the slides were counterstained with Mayer's hematoxylin, dehydrated, then mounted.

Positive control sections were done by using sections from tonsils. The staining specificity was checked on negative control slides by omitting the primary antibody.

#### Immunohistochemical Evaluation

The expression of GrzB was cytoplasmic and was seen in trophoblastic cells and stromal mesenchymal cells. Scoring was done for both intensity and percentage of expression for each slide.

The intensity of staining was graded from 0–3 as follows; no staining (0), weakly positive (1 +), moderately positive (2 +), strongly positive (3 +).

The percentage of expression of GrzB was determined on a scale from 0 to 100%. Then, the immunoreactivity score was calculated by multiplying the percentage and intensity. Images were obtained using a digital camera (Olympus, Tokyo, Japan).

### Flow Cytometry

Biopsy specimens were washed in PBS and then preserved in Rosewell Park Memorial Institute (RPMI) 1640 medium supplemented with 100 U/mL penicillin, 100 mg/mL streptomycin, and 5% fetal bovine serum (GIBCO BRL, Thermo Fisher Scientific, USA) until transfer to the laboratory. Tissue samples were then ground and digested by 3 ml collagenase enzyme type Ia and 2 ml PBS for 40 min at 37 °C in a shaking water bath. After centrifugation, the obtained cell suspensions were filtered, and the pellet was treated with red blood cell lysing solution and washed with PBS.

The processed placenta specimen and 100 µl of the whole blood sample were each incubated with 10 µl of fluoroisothiocyanate (FITC)-conjugated anti-TCR Vα7.2 [Clone: 3C10] (Beckman Coulter, France), phycoerythrin (PE)-conjugated anti-CD56 [Clone: B159], PE-cyanine 7 (PE-CY7)-conjugated anti-CD16 [Clone: 3G8] [Becton Dickinson (BD), USA], allophycocyanin (APC)-conjugated anti-CD161 [Clone: HP-3G10] (EXBIO, Czech Republic), APC-H7- conjugated anti-CD3 [Clone: SK7] (BD, USA) for 15 min at 4 °C in the dark. The lysing solution was then added to the mixture, and after centrifugation, the supernatant was discarded. Then after washing with PBS and centrifugation, the pellet was resuspended in PBS. An isotype-matched anti-human IgG was used with each sample as a negative control. Flow cytometric analysis was done by FACSCanto flow cytometer using FACS DIVA 7.0 software (BD, USA). A least 100,000 events were acquired per sample. Singlet events were gated by using a FSC-A plotted against a FSC-H. Then lymphocytes were identified in FSC-A SSC-A plot for further analysis, supplementary figure (S1). Afterwards, CD3^+^ lymphocytes were selected and TCR Vα7.2^+^ T cells were categorised based on CD161 relative expression into (CD3^+^Vα7.2 ^+^CD161^bright^) MAIT, (CD3^+^Vα7.2^+^CD161^dim^) MAIT-like and (CD3^+^Vα7.2 ^+^CD161^−^) non-MAIT cells. The expression of CD56 and CD16 was assessed within each Vα7.2^+^ T cell subset. In addition, The NKT cells (CD3^+^CD56^+^CD16^+^) were also measured within the CD3^+^ population. Gating on the CD3^−^ lymphocytes, the NK cell subsets and their expression of CD161 were evaluated (CD56^bright^CD16^bright^, CD56^bright^CD16^dim^, CD56^bright^CD16^−^, CD56^dim^CD16^bright^, CD56^dim^CD16^dim^ and CD56^dim^CD16^−^).

### Statistical Analysis

Continuous variables were presented as mean ± standard error (SE), whereas categorical data were presented as number (percentage). Results were analysed by IBM Statistical Package for the Social Sciences, version 25 (IBM SPSS statistics, USA). Normality of distribution was assessed using probability plots and the Shapiro–Wilk test. Independent sample t-test and Mann–Whitney U were used to compare variables among the different parametric or non-parametric groups, respectively. A Paired-T test was performed to evaluate the differences in cell frequencies between biopsy and blood samples. Using Kendall's tau and Spearman correlation coefficient, associations between variables were explored. A* p*-value was considered significant at less than 0.05.

## RESULTS

### Clinical and Laboratory Features of the Study Participants

The age of PAS patients matched that of controls (*p* = 0.7), whereas patients had younger gestational age than healthy controls (*p* < 0.0001). Higher parity was observed in PAS patients. Seventy-five percent of PAS patients had > 2 prior caesarian section deliveries compared to 15% in controls. About 53% and 25% of patients had history of previous abortions and evacuations, respectively. Placenta accreta was detected in approximately 92% of PAS cases, while placenta percreta was found in the remaining cases. Only 6.3% of PAS patients had a history of PAS in previous pregnancies. Results are presented in Table (1).

**Table 1:** Clinical and laboratory features of the study participants

	**Variable**	**Patients**	**Control**	***p-*value**
**Age**		31.2 ± 5	30.6 ± 6	0.7
**Gestational age**		35.9 ± 0.7	38.6 ± 0.5	< 0.0001**
**Number of CS**	0	1 (3.1%)	4 (20%)	< 0.0001**
	1	2 (6.3%)	12 (60%)	
	2	5 (15.6%)	1 (5%)	
	3	10 (31.3%)	1 (5%)	
	4	14 (43.7%)	1 (5%)	
	5	0 (0%)	1 (5%)	
**PAS in previous pregnancies**		2 (6.3%)	0	-
**PAS type**				
Accreta		29 (90.6%)	NA	-
percreta		3 (9.4%)		
**Complete blood count**				
Hb (gm/dl)		11.4 ± 1	11.6 ± 3	0.8
TLC (X10^3^/μl)		10.3 ± 4	9.3 ± 3	0.3
Neutrophil	%	70.3 ± 10	72.3 ± 8	0.4
	count	7.4 ± 3	7.4 ± 3	0.99
Lymphocytes	%	21.3 ± 8	22.5 ± 10	0.6
	count	2 ± 0.8	1.9 ± 0.6	0.4

Data presented as mean ± SE or number (percent %)

*CS* caesarian section, *PAS* placenta accreta spectrum, *Hb* hemoglobin, *TLC* total leukocyte count

*p-*value is *significant at < 0.05 and **highly significant at < 0.0001

### Placenta Levels of Angiogenic and Antiangiogenic Factors

**Fig. 1:**
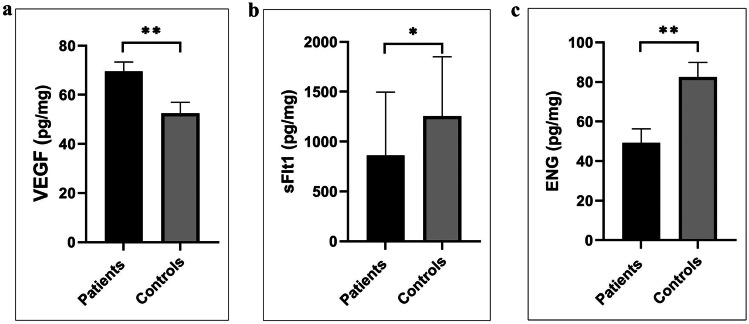
Placenta levels of **a** Human Vascular endothelial cell growth factor (VEGF), **b** Soluble FMS Like Tyrosine Kinase (sFLT-1), and **c** Endoglin (ENG) in PAS patients and healthy controls.

A significant rise was observed in the level of the angiogenic VEGF (69.6 ± 4 pg/mg vs. 52.5 ± 4 pg/mg, *p* = 0.006). In contrast, lower levels of anti-angiogenic sFLT-1 and ENG were detected in the placentae of PAS patients compared to healthy controls (861.9 ± 127 pg/mg vs. 1255.6 ± 149 pg/mg, *p* = 0.03, and 49.2 ± 7 pg/mg vs. 82.6 ± 7 pg/mg, *p* = 0.002, respectively), Fig. (1).

### Histopathologic Evaluation of Placenta in PAS Patients and Healthy Controls

**Fig. 2:**
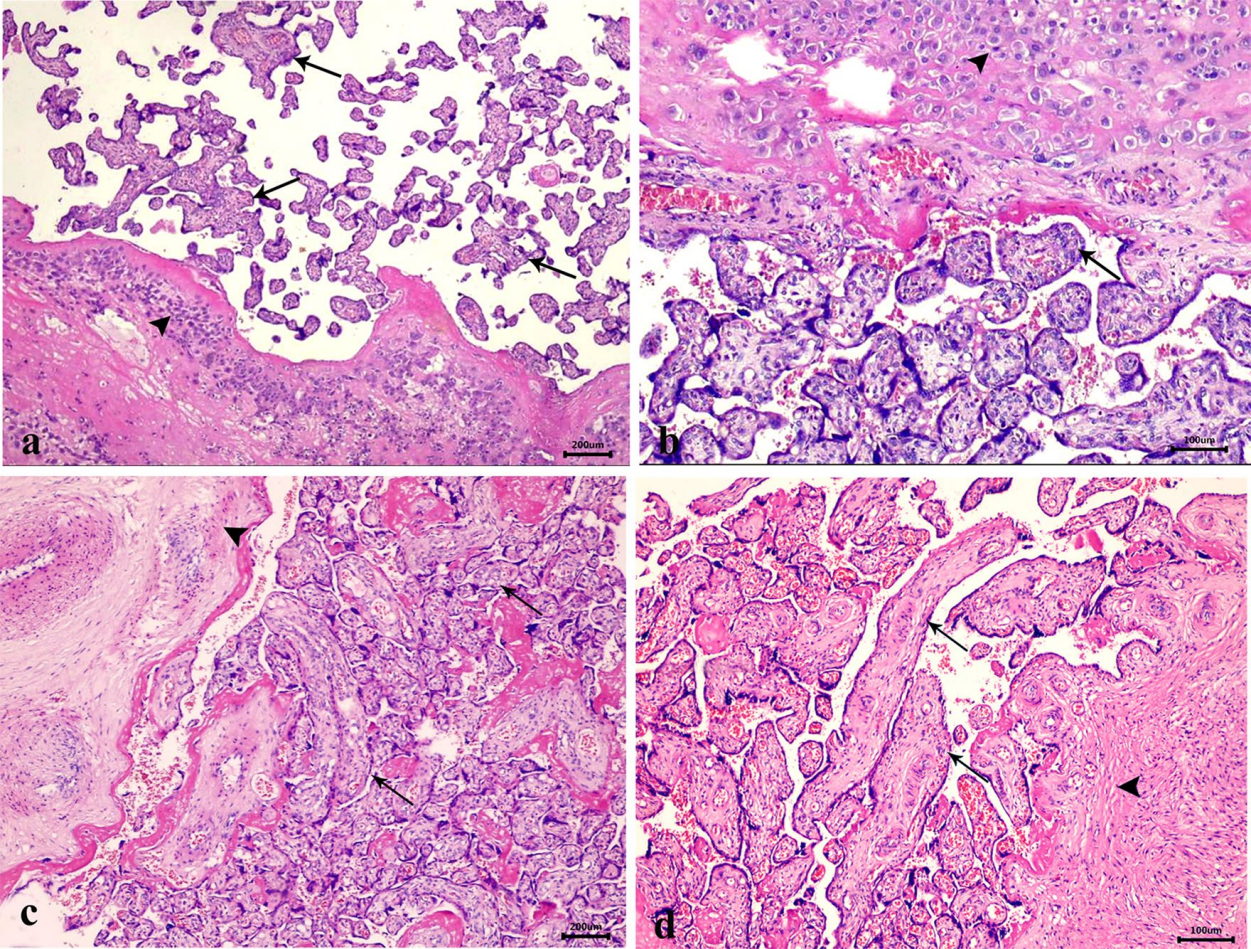
Hematoxylin and eosin-stained sections from **a**: normal placenta showing decidua (arrowhead) is present in the basal plate of chorionic villi. The chorionic villi are illustrated by (black arrows) (× 40). **b**: higher power of the previous slide showing large pale decidual cells (arrowhead) and chorionic villi invested by trophoblast cells and have a core of mesenchyme containing capillaries (black arrow) (× 100). **c**: placenta accreta showing chorionic villi (black arrows) lies directly on the fibrin layer, separating them from myometrium (arrowhead) (× 40). **d**: higher power of the previous slide showing the placental villi (black arrows) interdigitate directly with the uterine myometrium (arrowhead), without intervening decidual plate (× 100).

Sections of normal placenta stained with Hx & E showed decidua with clusters of large pale staining decidual cells present in the basal plate of chorionic villi. Chorionic villi also appeared invested by trophoblast cells and had a mesenchymal core containing capillaries (Fig. 2a, b). In contrast, the placenta accreta stained Hx & E sections revealed chorionic villi interdigitate directly with the uterine myometrium, without intervening decidual plate (Fig. 2c, d).

### Granzyme B Expression in the Placental Villous Trophoblast and Stroma of PAS Patients and Healthy Controls

**Fig. 3:**
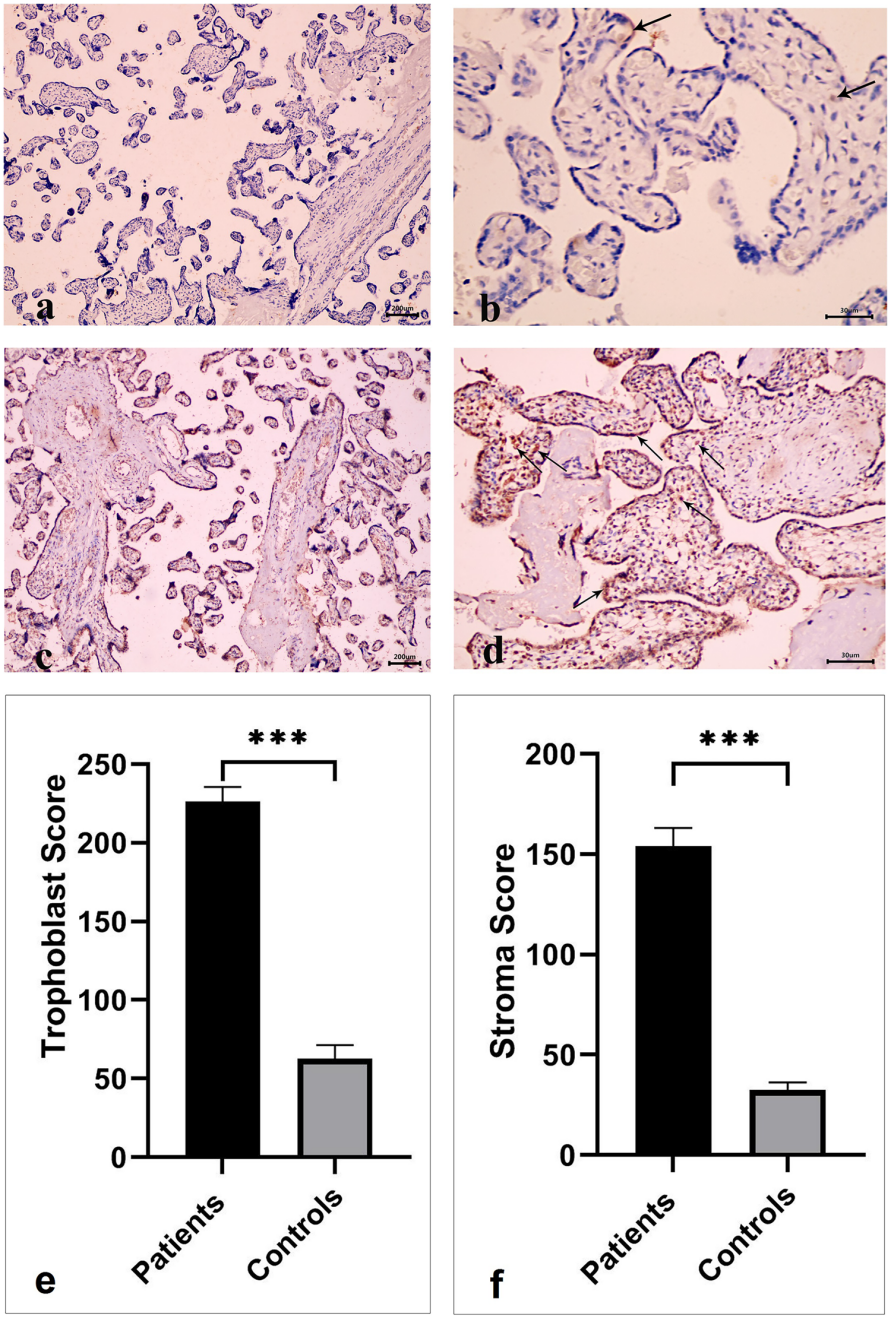
**a**, **b** Immunohistochemical staining of granzyme B (GrzB) in normal placenta showing only a few trophoblast cells and stromal cells of chorionic villi are positive (black arrows) (× 40, × 400 respectively). **c**, **d** Immunohistochemical staining of GrzB in placenta accreta showing positive cytoplasmic expression in many trophoblastic and chorionic villi stromal cells (black arrows) (× 40, × 400 respectively). **e**, **f** Trophoblast and stroma scores of the GrzB expression in PAS patients and healthy controls.

We also investigated the expression of GrzB using the immunohistochemical technique. We observed only a few positive trophoblast cells and stromal cells of chorionic villi in the normal placenta (Fig. 3a, b). On the contrary, placenta accreta showed positive cytoplasmic expression in many trophoblastic and chorionic villi stromal cells (Fig. 3c, d).

As illustrated in Fig. (3e, f), the immunohistochemical trophoblast and stroma scores of the GrzB expression were sharply increased in the PAS cases compared to the healthy control group (226.4 ± 9 vs. 62.8 ± 9, *p* < 0.0001 and 154 ± 9 vs. 32.5 ± 4, *p* < 0.0001). Twenty-six PAS patients (81%) had a GrzB trophoblast score greater than or equal to 165, while 24 patients (75%) had a GrzB stroma score greater than or equal to 100. In contrast, all women with normally implanted placentae showed low GrzB trophoblast and stroma scores (< 165 and < 100, respectively).

### Percentages of MAIT Cells, NK Cells, and NKT Cells in both Blood and Placenta of PAS Patients and Controls

**Fig. 4:**
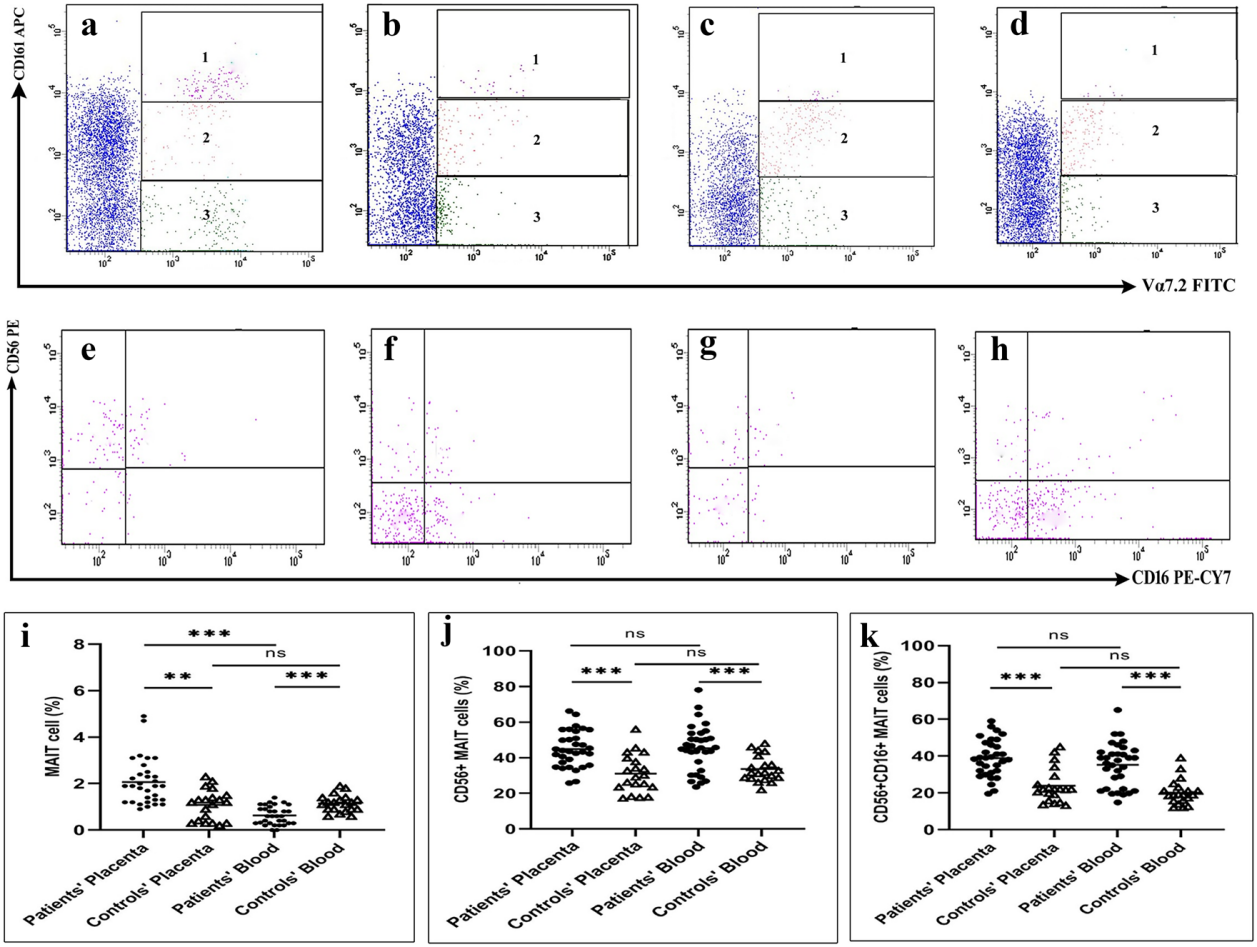
**a**, **b** Plots showing mucosal-associated invariant T (MAIT) cells in the placenta of PAS patients and healthy controls, respectively. **c**, **d** MAIT cells in the blood of PAS patients and healthy controls, respectively. **e**, **f** MAIT cells expressing CD56 and CD16 cells in the placenta of PAS patients and healthy controls, respectively. **g**, **h** MAIT cells expressing CD56 and CD16 cells in the blood of PAS patients and healthy controls, respectively. **i**–**k** Levels of MAIT cells, CD56^+^ MAIT cells, and CD56^+^CD16^+^ MAIT cells in PAS patients and healthy controls, respectively.

As shown in Fig. (4), MAIT cells were significantly higher in the decidua tissue of PAS patients than that in healthy controls (2 ± 0.2 vs. 1.1 ± 0.1, *p* = 0.001). Meanwhile, lower levels of MAIT cells were observed in patients' blood compared with the control group (0.63 ± 0.07 vs. 1.1 ± 0.08, *p* < 0.0001). Only in PAS patients, MAIT cells were considerably higher in decidua tissue than in blood (*p* < 0.0001). In addition, higher percentages of CD56^+^ MAIT cells were observed in patients' decidua and blood in comparison with the controls' decidua and blood, respectively (44.9 ± 2 vs. 31.3 ± 3, *p* < 0.0001 and 45.4 ± 2 vs. 33.7 ± 2, *p* < 0.0001). Also, a more significant proportion of MAIT cells were coexpressing CD56 and CD16 in patients' decidua and blood than in the controls' decidua and blood, respectively (38.9 ± 2 vs. 24.2 ± 2, *p* < 0.0001 and 35.2 ± 2 vs. 20.2 ± 2, *p* < 0.0001). Somewhat similar changes were observed in MAIT-like cells in decidua tissue but not in blood, and no significant differences were detected in non-MAIT cells in the decidua.

**Fig. 5:**
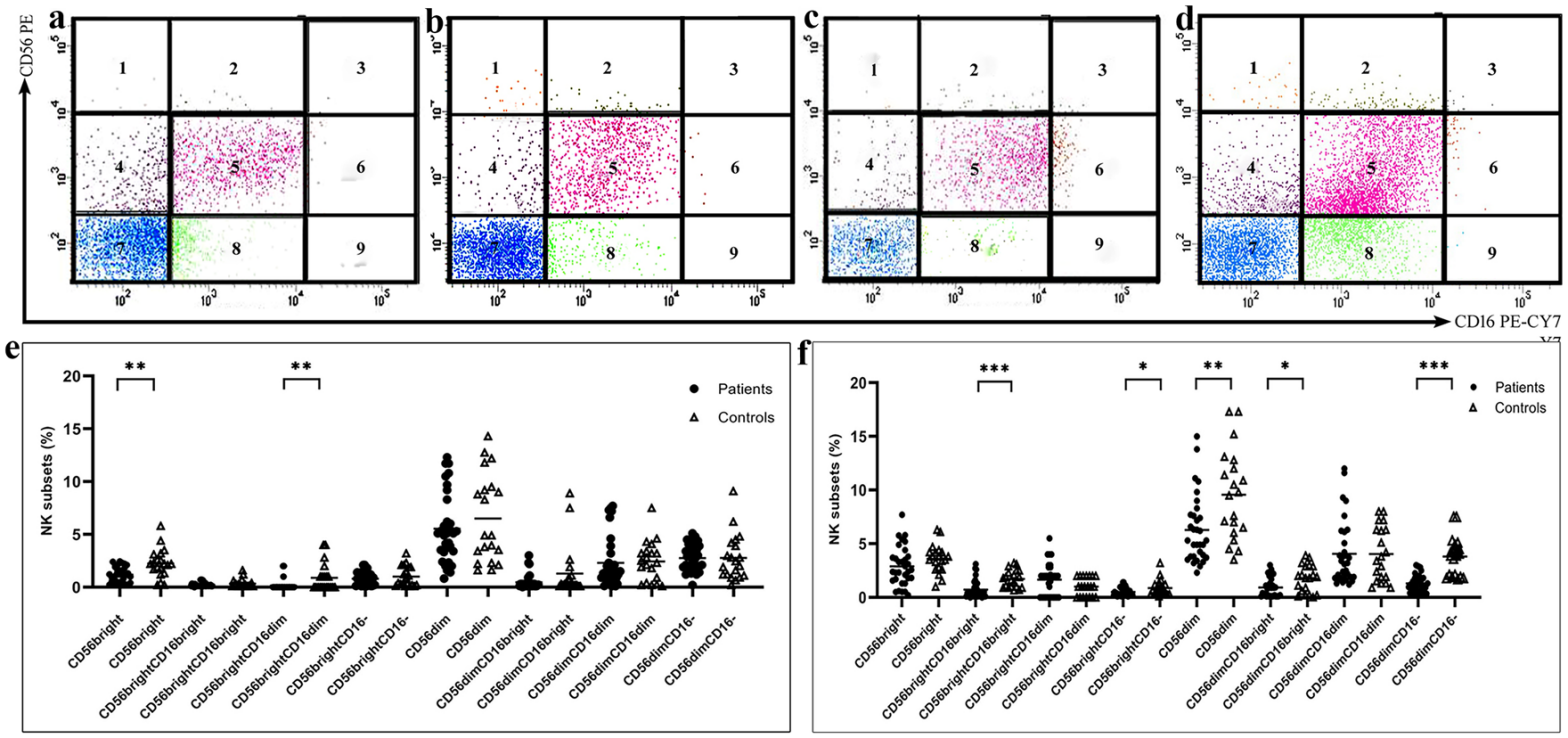
**a**, **b** Plots showing natural killer (NK) cell subsets in the placenta of PAS patients and healthy controls, respectively **c**, **d** NK cell subsets in the blood of PAS patients and healthy controls, respectively. Levels of NK cell subsets in **e** placenta and **f** blood of PAS patients and healthy controls.

Regarding NK cells (Fig. 5), the level of CD56^bright^ cells decreased significantly in decidua tissue of PAS patients than healthy controls, particularly the CD56^bright^CD16^dim^ subset. Moreover, CD161 expression levels by the CD56^bright^CD16^bright^ and CD56^bright^CD16^dim^ increased substantially. Meanwhile, CD56^bright^CD16^bright^ and CD56^bright^CD16^−^ NK cells decreased in the blood of PAS patients than healthy controls with higher CD161 expression by CD56^bright^CD16^−^ NK cells.

**Fig. 6:**
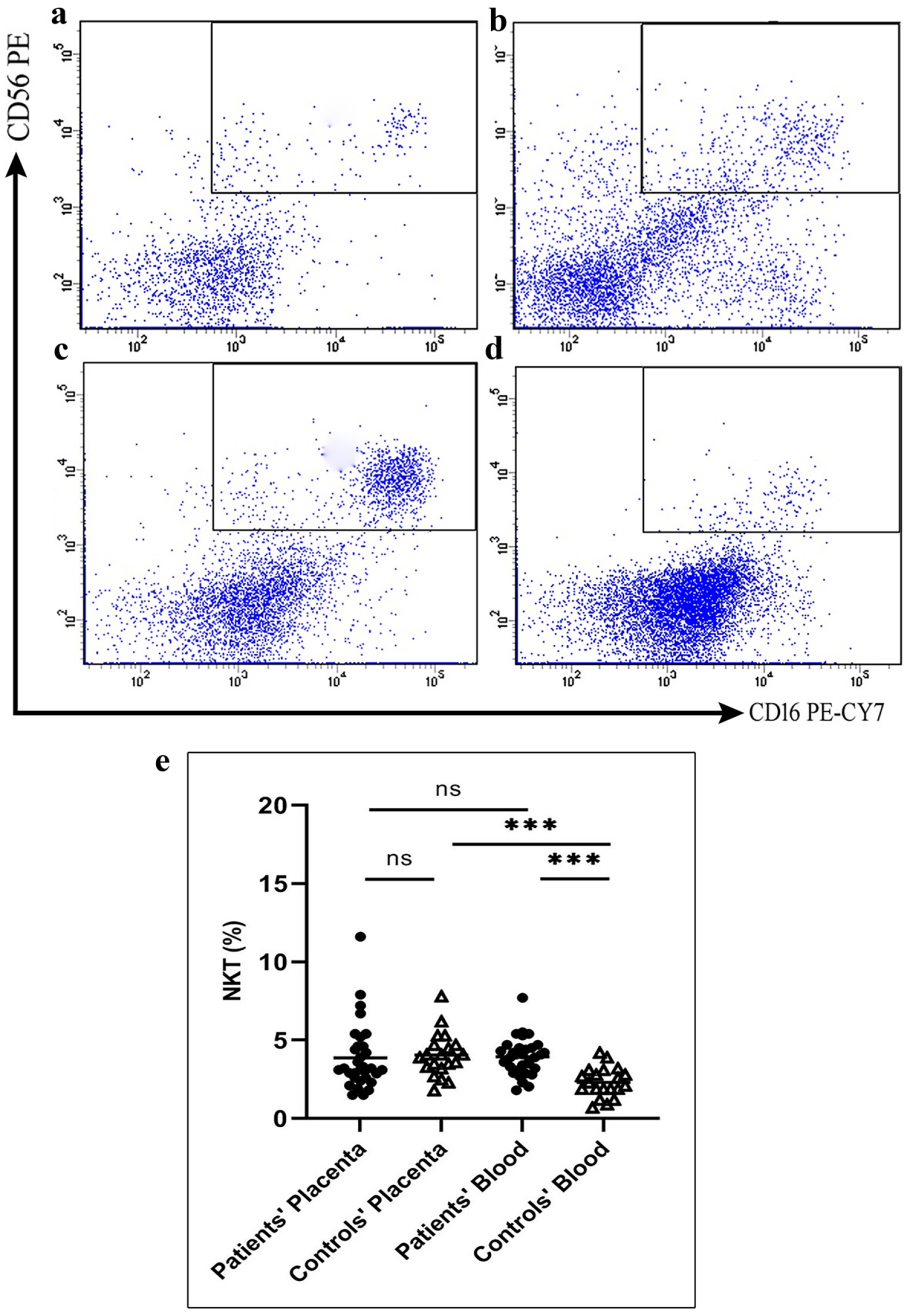
**a**, **b** Plots showing natural killer T (NKT) cell in placenta of PAS patients and healthy controls, respectively **c**, **d** NKT cell in the blood of PAS patients and healthy controls, respectively **e** Levels of NKT cell subsets in placenta and blood of PAS patients and healthy controls.

Even though no significant changes were observed in the CD56^dim^ NK cells in decidua tissue of PAS patients, a significant decrease was seen in their blood level in comparison with controls especially, the CD56^dim^CD16^bright^ and the CD56^dim^CD16^−^. A higher expression level of CD161 was detected among the CD56^dim^CD16^bright^ NK cells in the blood of PAS patients than controls. NKT cells increased only in the blood of PAS patients than controls, Fig. (6).

Percentages of MAIT cells, NK cells, and NKT cells in both blood and placenta of PAS patients and controls are shown in details in the supplementary table (S2).

### Correlations of MAIT, NK, and NKT Cells with Some PAS Risk Factors

Placenta MAIT cells were directly associated with maternal age (r = 0.3, *p* = 0.04), parity, and the number of previous caesarian sections (r = 0.3, *p* < 0.0001). Additionally, CD56^+^ MAIT cells, and particularly the CD56^+^CD16^+^ MAIT cells, were positively related to the number of prior cesarean sections (r = 0.4, *p* < 0.0001). In the same line, blood CD56^+^ MAIT cells and predominantly the CD56^+^CD16^+^ correlated directly with the number of previous abortions (r = 0.3, *p* < 0.0001) and evacuations (r = 0.4, *p* < 0.0001). Furthermore, blood CD56^+^CD16^+^ MAIT cells were directly associated with the number of prior cesarean sections (r = 0.3, *p* < 0.0001) and indirectly with the gestational age (r = –0.3, *p* < 0.0001). Also, blood CD56^+^ MAIT-like cells were positively associated with the gestational age (r = 0.3, *p* < 0.0001).

Decidua CD56^bright^CD16^dim^ NK cells correlated positively with the gestational age (r = 0.3, *p* < 0.0001). Moreover, CD161^+^CD56^bright^CD16^dim^ NK cells associated directly with parity and the number of previous cesarean sections (r = 0.3, *p* < 0.0001). Likewise, CD161^+^CD56^bright^CD16^bright^ NK cells correlated positively with the maternal age (r = 0.3, *p* = 0.008), number of previous abortions and cesarean sections (r = 0.3, *p* < 0.0001).

Blood CD56^bright^CD16^bright^ NK cells correlated positively with both the maternal and gestational age (r = 0.3, *p* = 0.02 and r = 0.3, *p* < 0.0001). CD56^dim^CD16^−^ NK cells correlated positively with gestational age (r = 0.3, *p* < 0.0001), as well, and negatively with the number of previous cesarean sections (r = –0.3, *p* < 0.0001). On the contrary, blood NKT cells were directly associated with the number of previous cesarean sections (r = 0.3, *p* < 0.0001).

### Correlations of MAIT, NK and and NKT Cells with the GrzB Staining Scores, Angiogenic and Antiangiogenic Factors

Decidua MAIT cells showed positive correlations with GrzB trophoblast and stroma scores (r = 0.4, *p* = 0.001, and r = 0.6, *p* < 0.0001, respectively). Besides, the CD56^+^ MAIT cells were inversely related to ENG level in placenta (r = –0.4, *p* = 0.008) and were directly related to the GrzB trophoblast and stroma scores (r = 0.4, *p* = 0.004, and r = 0.4, *p* = 0.003, respectively). Likewise, CD56^+^CD16^+^ MAIT cells correlated positively with the placenta VEGF level (r = 0.2, *p* = 0.04) and GrzB trophoblast and stroma scores (r = 0.5, *p* < 0.0001, and r = 0.4, *p* < 0.0001, respectively), and negatively with ENG and sFLT-1 placenta levels (r = –0.4, *p* = 0.004, and r = –0.4, *p* = 0.01, respectively).

On the contrary, blood MAIT cells showed negative relations with GrzB trophoblast and stroma scores (r = –0.4, *p* = 0.003, and r = –0.5, *p* < 0.0001, respectively) and a positive relation with ENG placenta level (r = 0.4, *p* = 0.002). Besides, the CD56^+^ MAIT cells correlated positively with GrzB trophoblast and stroma scores (r = 0.5, *p* < 0.0001) and inversely related to ENG level in placenta (r = –0.4, *p* = 0.009). Similarly, CD56^+^CD16^+^ MAIT cells correlated positively with the GrzB trophoblast and stroma scores (r = 0.6, *p* < 0.0001) and negatively with ENG and sFLT-1 placenta levels (r = –0.5, *p* = 0.001, and r = –0.3, *p* = 0.01, respectively).

Similarly, total decidua CD56^+^ MAIT-like and CD56^+^CD16^+^ MAIT-like cells showed inverse relations with sFLT-1 placenta level (r = –0.5, *p* = 0.001) and direct relations with the GrzB trophoblast score (r = 0.3, *p* = 0.02 and r = 0.3, *p* = 0.01, respectively). Also, CD56^+^CD16^+^ MAIT-like cells were indirectly related to ENG placenta level (r = –0.3, *p* = 0.045).

Blood non-MAIT cells revealed a negative correlation with sFLT-1 placenta level (r = –0.3, *p* = 0.01) and positive correlations with the GrzB trophoblast score (r = 0.4, *p* = 0.004 and r = 0.3, *p* = 0.01, respectively).

Placenta CD56^bright^ NK cells were negatively associated with the GrzB trophoblast and stroma scores (r = –0.4, *p* = 0.002 and r = –0.3, *p* = 0.01, respectively). CD56^bright^CD16^dim^ NK cells had a positive correlation with ENG placenta level (r = 0.3, *p* = 0.02) and inverse relations with the placenta VEGF level (r = –0.2, *p* = 0.046) and GrzB trophoblast and stroma scores (r = –0.5, *p* < 0.0001, and r = –0.4, *p* = 0.001, respectively). Moreover, CD161^+^CD56^bright^CD16^dim^ NK cells displayed positive correlations with the GrzB trophoblast and stroma scores (r = 0.3, *p* = 0.03, and r = 0.3, *p* = 0.007, respectively) and negative correlation with ENG placenta level (r = –0.3, *p* = 0.04). Likewise, CD161^+^CD56^bright^CD16^bright^ NK cells displayed positive correlations with the GrzB trophoblast and stroma scores (r = 0.3, *p* = 0.02) and negative correlation with sFLT-1 placenta level (r = –0.3, *p* = 0.03). CD56^bright^CD16^−^ NK cells had a negative correlation with ENG placenta level (r = –0.3, *p* = 0.04), while, CD161^+^CD56^bright^CD16^−^ NK cells correlated indirectly with sFLT-1 placenta level (r = –0.3, *p* = 0.03).

Blood CD56^bright^CD16^bright^ NK cells were directly related to sFLT-1 placenta level (r = 0.4, *p* = 0.003) and indirectly related to VEGF placenta level (r = –0.4, *p* = 0.001) and GrzB trophoblast and stroma scores (r = –0.5, *p* < 0.0001, and r = –0.4, *p* = 0.001, respectively). Moreover, CD56^bright^CD16^−^ NK cells had negative correlations with VEGF placenta level (r = –0.3, *p* = 0.01) and GrzB trophoblast score (r = –0.3, *p* = 0.03). CD161^+^CD56^bright^CD16^−^ NK cells were directly associated with VEGF placenta level (r = 0.3, *p* = 0.01) and indirectly associated with the sFLT-1 placenta level (r = –0.3, *p* = 0.04).

Blood total CD56^dim^ NK cells and CD56^dim^CD16^bright^ NK cells were negatively associated with the placenta VEGF level (r = –0.3, *p* = 0.02 and r = –0.3, *p* = 0.04, respectively), GrzB trophoblast score (r = –0.3, *p* = 0.01 and r = –0.3, *p* = 0.02, respectively) and stroma score (r = –0.3, *p* = 0.01) and had a positive correlation with ENG placenta level (r = 0.3, *p* = 0.02 and r = 0.4, *p* = 0.01, respectively). In addition, CD56^dim^CD16^−^ NK cells showed inverse relations with the placenta VEGF level (r = –0.3, *p* = 0.03) and GrzB trophoblast and stroma scores (r = –0.5, *p* < 0.0001, and r = –0.6, *p* < 0.0001, respectively) and a positive relation with ENG placenta level (r = 0.6, *p* < 0.0001).

NKT cells displayed positive correlations with the GrzB trophoblast and stroma scores (r = 0.5, *p* < 0.0001) and negative correlation with ENG placenta level (r = –0.4, *p* = 0.002).

### Correlations among the percentages of MAIT cells, NK cells, and NKT cells

Decidua MAIT cells correlated inversely with the CD56^bright^CD16^dim^ (r = –0.3, *p* = 0.02), and directly with CD161^+^CD56^bright^CD16^dim^ (r = 0.3, *p* = 0.02) and CD161^+^CD56^bright^CD16^bright^ NK cells (r = 0.3, *p* = 0.01). Also, decidua CD56^+^ MAIT cells displayed positive correlations with CD161^+^CD56^bright^CD16^dim^ (r = 0.4, *p* = 0.004) and CD161^+^CD56^bright^CD16^bright^ NK cells (r = 0.4, *p* = 0.003). Decidua CD56^+^CD16^+^ MAIT cells had an inverse relation with the CD56^bright^ NK cells (r = –0.4, *p* = 0.005), and direct relations with CD161^+^CD56^bright^CD16^bright^, CD161^+^CD56^bright^CD16^dim^ and CD161^+^CD56^bright^CD16^−^ NK cells (r = 0.4, *p* = 0.003, r = 0.3, *p* = 0.006, and r = 0.4, *p* = 0.003, respectively).

Blood MAIT cells showed positive correlations with CD56^bright^CD16^−^ and CD56^dim^CD16^−^ NK cells (r = 0.3, *p* = 0.03, and r = 0.6, *p* < 0.0001, respectively). CD56^+^ MAIT cells displayed a positive correlation with CD56^dim^CD16^bright^ NK cells (r = 0.3, *p* = 0.007). Likewise, CD56^+^CD16^+^ MAIT cells had inverse relations with the CD56^bright^CD16^bright^, CD56^dim^CD16^bright^ and CD56^dim^CD16^−^ NK cells (r = –0.3, *p* = 0.007, r = –0.4, *p* = 0.003, and r = –0.4, *p* = 0.002, respectively), and direct relations with CD161^+^CD56^bright^CD16^−^ and CD161^+^CD56^dim^CD16^bright^ NK cells (r = 0.3, *p* = 0.03, and r = 0.3, *p* = 0.008).

Blood NKT cells showed a negative correlation with blood MAIT cells (r = –0.4, *p* = 0.001) and positive correlations with placenta MAIT cells (r = 0.3, *p* = 0.03), blood and placenta CD56^+^ MAIT cells (r = 0.4, *p* = 0.002, and r = 0.5, *p* < 0.0001, respectively) and blood and placenta CD56^+^CD16^+^ MAIT cells (r = 0.5, *p* < 0.0001). Also blood NKT showed direct relations with blood CD56^bright^CD16^dim^ (r = 0.3, *p* = 0.03), CD161^+^CD56^bright^CD16^−^ (r = 0.4, *p* = 0.001) and CD161^+^CD56^dim^CD16^bright^ NK cells (r = 0.3, *p* = 0.02), in addition to inverse relations with CD56^bright^CD16^bright^ (r = –0.5, *p* < 0.0001), CD56^bright^CD16^−^ (r = –0.3, *p* = 0.01) and CD56^dim^CD16^−^ NK cells (r = –0.6, *p* < 0.0001).

## DISCUSSION

The PAS pathogenesis is still not known with certainty [21]. In addition, data regarding immune dysregulation in PAS is still lacking. This is the first study analysing alterations in NK cell subsets, NKT and MAIT cells in placenta and blood of PAS patients and correlating these disturbances with the modulation in the expression of some angiogenic and antiangiogenic factors implicated in trophoblast invasion and with GrzB distribution in trophoblast and stroma.

The fine coordination among placental angiogenic factors as VEGF and antiangiogenic factors as sFLT-1 and ENG is essential for developing normal placenta and trophoblast invasion. Authors reported that in human placentas, the expression of VEGF-A correlates with trophoblast invasion, and blocking its signaling inhibits cytotrophoblast invasion [22]. Likewise, McMahon and colleagues have shown the association between low expression of the sFLT-1 protein, an endogenous inhibitor of VEGF**,** at the maternal–fetal interface and invasive placenta compared to the normal one [23]. ENG is a TGF-*β* receptor ligand that disturbs the TGF-*β* mediated signaling, and its loss was associated with EVT increased invasive ability [24, 25]. In accordance with the previous findings, we detected a significantly increased expression of VEGF associated with decreased expression of antiangiogenic sFLT-1 and ENG in placentae of PAS patients compared to healthy controls.

Granzyme B (GrzB) is a serine protease produced by different cells and has multifunctional proinflammatory effects. It is also involved in several pathological inflammatory diseases [26]. Cytokines and extracellular matrix (ECM) components are GrzB extracellular substrates [27]. GrzB can process and activate pro-fibrotic, proinflammatory, and aging factors as the IL-1α and IL-18 cytokines [27]. Increased GrzB levels were found in inflamed tissues like atherosclerotic plaque in cardiovascular diseases and circulation during inflammation (e.g., rheumatoid arthritis) [28–30].

Matrix remodeling proteases and their inhibitors are fundamental during parturition in the term placenta. Hirst and others detected GrzB expression in term placenta and the testis in the absence of perforin, signifying a non-cytotoxic role of GrzB in reproduction. Their results from immunohistochemical and RT–PCR analysis of term placentae reported the presence of GrzB in syncytial trophoblasts suggesting that GrzB may contribute to trophoblastic invasion and extracellular matrix remodeling during parturition [28]. In the same line, our results showed that the immunohistochemical trophoblast and stroma scores of the GrzB expression increased sharply in the PAS cases compared to the controls.

In non-pregnant women, MAIT cells are present in the endometrium and cervix, but peripheral blood contains higher frequencies of MAIT cells among CD3^+^ T cells than the endometrium [29]. In early pregnancy, MAIT cells are present in the decidua [30]. However, there is no information about their phenotype, relative abundance, or location [17]. At term pregnancy, MAIT cells accumulate in the maternal blood that flows into the intervillous space inside the placenta. The proportion of MAIT cells in peripheral blood is similar to term pregnancy decidua parietalis. However, the decidua basalis is richer in MAIT cells than the decidua parietalis [11]. In response to riboflavin-producing *Escherichia coli,* MAIT cells exhibit a stronger IFN-γ and GrzB expression in the placenta than paired peripheral MAIT cells [9]. By far, evidence supporting the role of MAIT cells in pregnancy complications has been minimal. However, a recent study pointed to a possible relation between MAIT cells and recurrent spontaneous miscarriage. Authors in the latter research proposed an association between activation of MAIT cells and maternal inflammation that may affect fetal survival [31].

Our results showed that MAIT cells increased in the placenta tissue and decreased in the blood of PAS patients than in healthy controls. Only in PAS patients, MAIT cells were considerably higher in placenta tissue than in blood. Higher percentages of total CD56^+^ and CD56^+^CD16^+^ MAIT cells were observed in patients' placenta and blood compared to the controls' placenta and blood, respectively. Somewhat similar changes were observed in MAIT-like cells in placenta tissue but not in blood. The changes in MAIT cell distribution may indicate the migration of more cells from blood to the placenta, suggesting a possible role of these cells in the pathogenesis of PAS. Besides, the higher level of non-MAIT cells in the blood of PAS patients may designate that the reduction in peripheral MAIT cells seems to be due, at least in part, to a loss in CD161 expression and not merely the result of trafficking into mucosal tissues, as was deduced earlier [32]. Likewise, the increase in MAIT-like cells in the placenta that, unlike MAIT cells, was not accompanied by a decrease in its level in blood may be attributable to down-regulation of MAIT cell CD161 following activation in the placenta.

The association of MAIT cells and PAS was supported by the direct correlations that we observed between placenta MAIT cells, total CD56^+^ MAIT cells, and particularly the CD56^+^CD16^+^ MAIT cells with several PAS risk factors, including parity and the number of previous caesarian sections. Also, these cells showed positive correlations with GrzB trophoblast and stroma scores, and CD56^+^CD16^+^ MAIT cells correlated positively with the placenta VEGF level and negatively with ENG and sFLT-1 placenta levels. Unlike blood MAIT cells, comparable correlations were observed in CD56^+^ and CD56^+^CD16^+^ MAIT cells in the blood.

Lately, Haliloglu and coauthors reported an increase in MAIT cell total number in the cord blood of nine PAS patients compared with healthy controls. However, they didn’t detect significant differences in TNF-α, IFN-γ cytokines and GrzB production by MAIT cells between the PAS patients and healthy controls, proposing an increase in MAIT cell number but not activity in PAS patients [33].

Recent studies reported an association between MAIT cells and microbiota [18, 19]. Possible involvement of dysbiosis in PAS was previously suggested [8]. Analysis of MAIT cell relation with dysbiosis may open new avenues in understanding PAS development. Still, the mechanistic role of MAIT and particularly CD56^+^CD16^+^ MAIT in PAS pathogenesis remains to be determined. We propose some mechanisms that may explain the possible role of MAIT cells in PAS pathogenesis. The first mechanism may be through their relations with angiogenic and antiangiogenic factors affecting trophoblast invasion, as was detected in our results. A previous study revealed that *in-vitro* stimulation of human MAIT cells by 5-OP-RU, akin to mouse MAIT cell experimental activation with *Legionella*, resulted in striking upregulation of proinflammatory gene as well as genes related to tissue repair such as platelet-derived growth factor subunit B, transforming growth factor beta-1, matrix metallopeptidase, and angiogenesis such as VEGF, granulocyte–macrophage colony-stimulating factor and hypoxia-inducible factor 1 subunit alpha [34].

The second possible mechanism is maybe through their expression of GrzB or induction of GrzB production by other cells as trophoblast and stromal cells, which may have a proinflammatory role in augmenting inflammation that may ultimately lead to invasive placentation. Also, the cytokines produced by MAIT cells, such as TNF-α, IFN-γ, transforming growth factor-β (TGF-β), and IL-17 [15, 35–37], were found to have a potential role in regulating trophoblast invasion either directly or through bystander activation of other immune cells [38–40]. Moreover, MAIT cells were shown to promote activation of other immune cells, including NK cells [41].

The CD56-expressing MAIT cells have high expression levels of IL-12R and IL-18R and higher levels of the transcription factors PLZF, Eomes, and T-bet and, hence, have a high capacity to respond to IL-12 and IL-18 than their negative counterparts [42]. Furthermore, CD56^+^ MAIT cells' responsiveness to innate cytokines makes them more competent in mounting MR1-independent responses during viral, bacterial, and sterile inflammatory conditions [43, 44]. Although CD16 was demonstrated to be the most potent activating receptor on freshly isolated human NK cells having strong cytotoxicity and cytokine production properties [45], still the CD16^+^ fraction of MAIT cells has not been fully characterized.

Intense research efforts have shed light on the role of NK cells in maintenance of pregnancy and the NK relation with pregnancy complications [46]. So far, little is known about NK subset distribution in placenta and blood of PAS patients. Decidual NK (dNK) cells produce multiple factors such as high levels of IL8 and CXCL10 that promote migration of primary cytotrophoblasts, angiogenic factors such as VEGF-A, VEGF-C and PGF [47]. In addition, they produce TGF-β1, TNF, and IFN-γ that inhibit migration and invasion of trophoblast [48–50]. Receptors for many of these molecules are expressed on primary EVTs [47].

Studies have shown that the number of peripheral and decidual NK cells decreases in the third trimester of pregnancy [51], with increasing CD56^dim^CD16^+^ than CD56^bright^CD16^−^ NK cells in decidua basalis [52]. Our results collectively showed decreased CD56^bright^ NK cells in the placenta and CD56^dim^ NK cells in blood with higher CD161 expression in PAS patients than in controls. Moreover, these subsets, especially the CD56^bright^CD16^dim^ NK cells in the placenta and the CD56^bright^CD16^−^ and CD56^dim^CD16^bright/−^ NK cells in the blood, showed several significant correlations with the PAS risk factors, angiogenic and antiangiogenic factors, and GrzB trophoblast and stroma scores, indicating the impact of these changes in the pathogenesis of PAS.

Laban and others reported an association between low CD56^bright^ dNK score in the placenta and the presence of morbidly adherent placenta accreta with an inverse correlation between dNK cell density and the degree of EVT invasion. The scanty dNK cells allow the deep invasion of the uterine tissue by the EVT [53]. A previous study also demonstrated that patients who had a history of previous cesarean section with normal placentation have a high density of the CD56^+^ cells in the scar tissue compared to deciduas obtained from patients who underwent elective cesarean sections without previous scar [54].

Natural killer T (NKT) cells are a distinct subtype of peripheral leukocytes. Upon activation, they secrete Th1 and Th2 cytokines and enhance the immune response through interactions with other immune cells, including conventional CD4^+^ T and CD8^+^ T cells, Tregs, macrophages, dendritic cells, NK cells, B cells and neutrophils [55], as well as their cytotoxic activities [56].

Invariant NKT (iNKT) cells are also abundant in the decidua compared to peripheral blood [57]. For normal placentation and implantation, EVT cells express CD1d and probably interact with maternal iNKT cells during the first trimester [58]. In addition, NKT cells can stimulate innate immune responses by quick cytokine production. This could elucidate their increased presence in placental and decidual tissues [59].

In preeclampsia, the increased decidual iNKT activity was associated with poor trophoblasts invasion of the spiral arteries and placental insufficiency, even though later events comprise a systemic inflammatory response [58]. So far, data regarding the changes in NKT in PAS are insufficient. We detected a rise in NKT cells exclusively in the blood of PAS patients than controls. NKT cells were also directly associated with the number of previous cesarean sections, GrzB trophoblast, and stroma scores, and inversely with ENG placenta level, proposing mostly an indirect contribution to PAS. The correlations detected among the MAIT cells, NK cells, and NKT cells together with the formerly mentioned correlations propose an interplay between these cells in the pathogenesis of PAS.

## CONCLUSION

Taken altogether, this is the first study analyzing the interrelation between MAIT cells, NK, and NKT in PAS. It seems that a cross-talk between these cell populations is somehow implicated in the pathogenesis of PAS. Assessment of MAIT cell impact on implantation and relation with dysbiosis in PAS may add a lot to our knowledge about PAS development.

## Supplementary Information

Below is the link to the electronic supplementary material.

Supplementary file1 (PDF 245 KB)

## Data Availability

All data generated or analyzed during this study are included in this published article.
