# Supplementary material for: Cross-Talk between Mucosal-Associated Invariant T, Natural Killer, and Natural Killer T Cell Populations is Implicated in the Pathogenesis of Placenta Accreta Spectrum
Source: Inflammation. 2023 Mar 31;46(4):1192–208. doi: 10.1007/s10753-023-01799-1 (PMC10359234; doi:10.1007/s10753-023-01799-1)
Supplement: Supplementary file 1 — Supplementary file1 (PDF 245 KB) [file 10753_2023_1799_MOESM1_ESM.pdf]

Supplementary Figure (1): (a) Singlet events were gated by using FSC-A plotted against FSC-H. (b) Lymphocytes were then identified in FSC-A SSC-A plot for further analysis.

a **b**

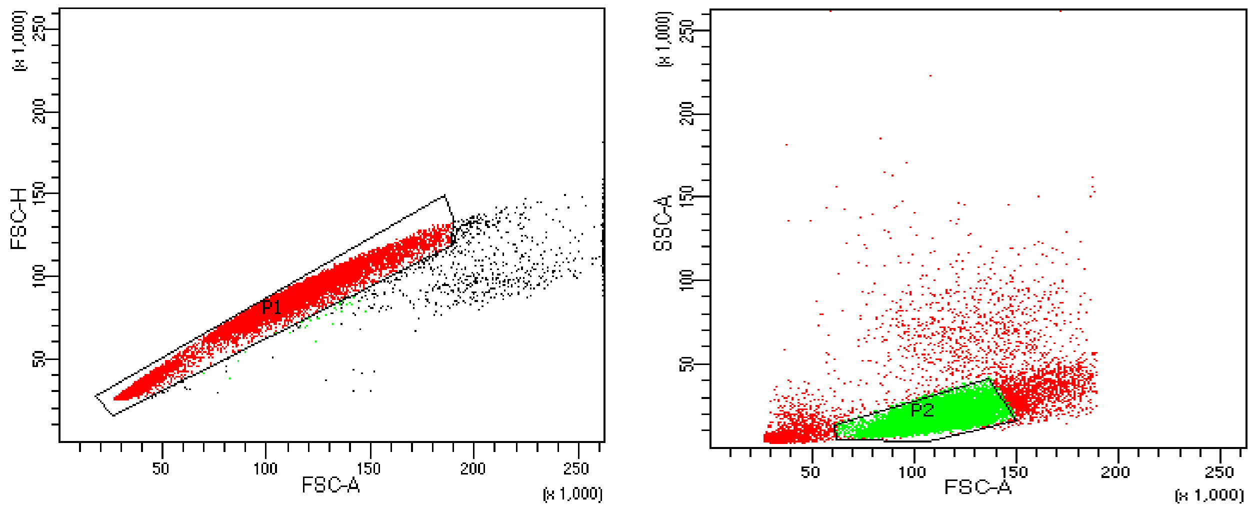

Supplementary table (S2): Percentages of MAIT cells, NK cells, and NKT cells in both blood and placenta of PAS patients and controls

Cells	Placenta			Blood			patients	control
	Patients	Control	*p1*-value	Patients	Control	*p2*-value	*p3*-value	*p4*-value
$CD3^+V\alpha7.2\ ^+CD161^{bright}$ MAIT	2±0.2	1±0.1	**0.001**	0.63±0.07	1.1±0.08	**<0.0001**	**<0.0001**	0.8
56^+MAIT	44.9±2	31.3±	**<0.0001**	45.4±2	33.7±2	**<0.0001**	0.9	0.4
56^+16^+MAIT	38.9±2	24±2	**<0.0001**	35.2±2	20.2±2	**<0.0001**	0.2	0.1
$CD3^+V\alpha7.2\ ^+CD161^{dim}$ MAIT-like	4.6±0.5	2.9±0.4	**0.03**	4±0.4	3.4±0.3	0.3	0.4	0.4
56^+MAIT-like	23.7±1.1	18.2±3	**0.04**	8±1	11.6±1	0.1	**<0.0001**	**0.01**
56^+16^+MAIT-like	19.9±1	14.9±2	0.06	5.6±1	7.2±1	0.07	**<0.0001**	**0.004**
$CD3^+V\alpha7.2\ ^+CD161^-$Non-MAIT	6±0.4	5.3±0.7	0.4	5.3±0.5	3±0.6	**0.02**	0.2	**0.03**
56^+non-MAIT	15.8±1	16.9±2	0.7	7.4±9.2	9.2±0.7	0.1	**<0.0001**	**0.002**
56^+16^+non-MAIT	11.9±1	13±2	0.98	4.3±0.7	4.8±0.4	0.07	**<0.0001**	**0.01**
$CD56\ ^{bright}$	1.2±0.1	2.3±0.3	**0.002**	2.9±0.3	3.6±0.3	0.2	**<0.0001**	**0.005**
$CD56\ ^{bright}CD16\ ^{bright}$	0.2±0.04	0.4±0.09	0.3	0.7±0.2	1.7±0.2	**<0.0001**	**<0.0001**	**<0.0001**
$CD161^+CD56^{bright}CD16^{bright}$	48.7±3	37.9±4	**0.02**	66.4±3	62.2±4	0.3	**<0.0001**	**0.001**
$CD56\ ^{bright}CD16^{dim}$	0.2±0.09	0.9±0.3	**0.004**	1.7±0.3	1±0.2	0.06	**<0.0001**	0.5
$CD161^+CD56\ ^{bright}CD16^{dim}$	49.7±3	38.7±4	**0.02**	60.8±3	55.7±3	0.2	**0.002**	**<0.0001**
$CD56^{bright}CD16^-$	0.8±0.1	1±0.2	0.6	0.5±0.07	0.9±0.2	**0.03**	**0.045**	0.9
$CD161^+CD56\ ^{bright}CD16^-$	51.3±4	48±5	0.6	61.8±3	47.3±3	**0.002**	**0.02**	0.9
$CD56\ ^{dim}$	5.5±0.6	6.6±1	0.6	6.2±0.6	9.6±0.9	**0.004**	0.2	**0.03**
$CD56\ ^{dim}CD16^{bright}$	0.5±0.1	1.3±0.6	0.6	0.9±0.2	1.7±0.3	**0.03**	0.06	0.1
$CD161^+CD56^{dim}CD16^{bright}$	60.2±4	64±4	0.5	68.7±3	57±4	**0.01**	0.05	0.1
$CD56\ ^{dim}CD16^{dim}$	2.3±0.4	2.5±0.4	0.3	4±0.5	4±0.6	0.9	**0.04**	**0.048**
$CD161^+CD56\ ^{dim}CD16^{dim}$	64±3	59.9±4	0.5	50.6±4	55.5±3	0.3	**0.002**	0.4
$CD56\ ^{dim}CD16^-$	2.8±0.2	2.9±0.5	0.5	1.3±0.1	3.8±0.4	**<0.0001**	**<0.0001**	0.1
$CD161^+CD56^{dim}CD16^-$	61.9±3	58.7±4	0.6	46.8±3	42±4	0.4	**0.002**	**0.003**
NKT	3.9±0.4	4±0.3	0.2	4±0.2	2.3±0.2	**<0.0001**	0.8	**<0.0001**
$CD161^+$NKT	48±3	45.9±3	0.6	51.4±3	59.8±4	0.7	0.3	**0.03**

P1-value: between placenta of patients and controls
p2-value: between blood of patients and controls
p3-value: between placenta and blood of patients
p4-value: between placenta and blood of controls
